# Ozonated water to treat pericoronitis - insights from a randomized triple-blind pilot trial

**DOI:** 10.1186/s12903-025-06652-5

**Published:** 2025-07-28

**Authors:** Célio Leone Ferreira Soares, Dhelfeson Willya Douglas-de-Oliveira, Cássio Roberto Rocha dos Santos, Olga Dumont Flecha, José Cristiano Ramos Glória, Patricia Furtado Gonçalves

**Affiliations:** Department of Dentistry, Federal University of Jequitinhonha and Mucuri Valleys, Rua da Glória, 187, Centro, Diamantina, Minas Gerais 39100-000 Brazil

**Keywords:** Pericoronitis, Third molar, Pain, Quality of life, Ozonated water

## Abstract

**Background:**

Pericoronitis, a painful inflammation of the soft tissues around a partially erupted tooth, is generally treated with debridement and saline irrigation. Ozone’s antimicrobial and anti-inflammatory properties make it a promising adjunctive therapy.

**Aim:**

To evaluate the clinical effects and impact on quality of life of treating pericoronitis in lower third molars using ozonated water compared to physiological saline solution.

**Materials and methods:**

This study involved patients with pericoronitis in lower third molars, following CONSORT guidelines. Ethical approval was obtained from the UFVJM Research Ethics Committee (protocol number 5.922.185). Patients were divided into two groups: irrigation with ozonated water (OZO, *n* = 5) or saline solution (SAL, *n* = 5). The pain was assessed using a visual analog scale, and quality of life was evaluated using OHIP-14, OHIP14-PD Br, and SF-36 questionnaires. Evaluations were conducted at baseline, 24 h, and 3, 7, 15, and 30 days post-treatment. Clinical parameters assessed included probing depth (PD), bone crest level (BCL), mouth opening (MO), and extent of edema/erythema (EEE) in the pericoronal hood region. Plaque Index (PI), Bleeding Index on Probing (BOP), and lower third molar positioning were also evaluated using panoramic radiography.

**Results:**

Intra-group analysis 7 days after treatment showed a significant reduction in pain (SAL: *p* = 0.018; OZO: *p* = 0.002) and the extent of edema/erythema (SAL: *p* = 0.002). OHIP-14 scores indicated significant intra-group differences at 30 days post-treatment (SAL: *p* = 0.043; OZO: *p* = 0.041).

**Conclusion:**

Ozonated water demonstrated similar efficacy to saline solution in managing pericoronitis, suggesting its potential as a viable alternative treatment.

**Clinical relevance:**

This study presents an alternative treatment option for pericoronitis.

**Clinical trial register:**

This clinical trial was registered at the Brazilian Clinical Trials Registry (ReBec) under protocol RBR-79pss6w (http://www.ensaiosclinicos.gov.br/rg/RBR-79pss6w/), Date of registration: 22/11/2024.

**Supplementary Information:**

The online version contains supplementary material available at 10.1186/s12903-025-06652-5.

## Introduction

Pericoronitis is a painful inflammatory condition commonly associated with erupting lower third molars in young adults, characterized by a complex anaerobic microbial flora [1–3]. Symptoms include pain and swelling in the pseudopocket around the tooth crown. Acute pericoronitis is treated as a dental emergency with local debridement and saline irrigation, sometimes supplemented with antibiotics and analgesics [4–6].

As a consequence of pain, oral health problems have been increasingly recognized as having a negative impact on patient’s quality of life and daily performance. There is also a need to investigate the impact on the quality of life of treated cases of pericoronitis through new clinical trials [7, 8]. Some products with anti-inflammatory and antimicrobial properties, in addition to debridement and local irrigation with saline solution, have been investigated as adjuvants in the treatment of pericoronitis. Among them, ozone, a natural compound with antimicrobial, anti-inflammatory, antifungal, immunomodulatory, and even analgesic effects against microorganisms, has been studied [9–11].

Studies have confirmed the efficacy of ozone in killing gram-negative and gram-positive microorganisms in ozonated water [12], as well as inhibiting the accumulation of experimental biofilm in vitro and being effective against bacteria in dental biofilm. It also reduces post-surgical infectious complications when used as an irrigation agent during the extraction of third molars [13].

This study investigates whether irrigation with ozonated water, compared to saline solution, improves the quality of life and clinical parameters in patients with pericoronitis. Given ozone’s antimicrobial and anti-inflammatory properties, we hypothesize that ozonated water will yield superior results in reducing symptoms and enhancing patient quality of life.

## Materials and methods

### Study design

This pilot randomized triple-blind clinical trial included two groups of five patients each, with a 30-day follow-up period. The study was approved by the Research Ethics Committee (protocol #5.922.185) of the Federal University of Jequitinhonha and Mucuri Valleys (UFVJM) and was conducted following CONSORT and according to the Helsinki Declaration. This clinical trial was registered at the Brazilian Clinical Trials Registry (http://www.ensaiosclinicos.gov.br/rg/RBR-79pss6w/) on 22/11/2024.

### Participants

Patients with symptomatic pericoronitis were recruited at the UFVJM Surgery and Periodontics Clinic from November 2024 to December 2024. Inclusion criteria were patients aged 18 years or older with periodontal health or periodontal status level I and II [14], presenting with spontaneous pain around the lower third molar. Exclusion criteria included recent antibiotic therapy, medical contraindications for periodontal probing, patients with systemic illnesses, or contraindications for analgesics. All participants received written informed consent and were included only after agreeing and signing it.

### Settings

This study was carried out at the Surgery and Periodontics Clinic of the UFVJM. A researcher (CLFS) was responsible for carrying out all evaluations, properly calibrated and trained with panoramic radiographs to assess the position of the third molar, and the evaluation of other clinical parameters, including the use of the Florida Probe, through test-retest with a 15-day interval. The intraclass correlation coefficient was kappa of 0.9. Each patient was evaluated in six appointments: baseline, 24 h, 3, 7, 15, and 30 days after the interventions. Another researcher (PFG) was responsible for performing the treatments and providing guidance to the patients.

### Randomization and blinding

This triple-blind trial used identical packaging for the liquids, which were transparent, odorless, and tasteless. An independent researcher conducted the randomization and sealed the intervention assignments in opaque envelopes. Patients and researchers remained blinded to the interventions.

One researcher received a standardized, unlabeled irrigation syringe during the procedure. Patients could not distinguish the product, as neither had taste, odor, or color. The evaluator was blinded to the treatment type, which was only revealed after the final statistical analysis.

### Interventions

Patients were treated with debridement under a pericironal hood using curettes and under local anesthesia with 2% lidocaine hydrochloride with 1:100,000 epinephrine (Lidocaine 2% DFL ^®^). The test group received ozonated water irrigation, and the control group received saline solution irrigation, both with a standard volume of 20 mL. Both groups received oral hygiene instructions and a standardized oral hygiene kit.

The ozonated water was produced chairside from MilliQ water, using an ozone generator model MedPlus V (Philozon^®^, Santa Catarina, Brazil), coupled with a glass column with a catalyst and microbubble diffuser in a stainless steel tube. The ozone generator was set to 60 µg/mL for five continuous minutes of bubbling in 250 mL of MilliQ water.

### Evaluated parameters

The study assessed multiple parameters using specific instruments at various time points. The primary outcomes are pain and quality of life, while the secondary outcomes include other clinical parameters. Pain was measured with the Visual Analog Scale (VAS) [15] and mouth opening with a millimeter ruler [16] at baseline and at 1, 3, 7, 15, and 30 days post-treatment. Edema and erythema were evaluated using dental floss and a millimeter rule [16] at the same intervals. Quality of life was assessed with Oral Health Impact Profile-14 [17], Oral Health Impact Profile-14 Periodontal Disease-Br [18], and Short Form-36 [19] at baseline and 30 days. Plaque index [20], bleeding on probing (WHO probe) [21], and probing depth (Florida probe) [22] were measured at baseline, 7, 15, and 30 days. Bone crest height (Williams Probe) [22] was recorded at baseline and 30 days, while the third molar position was assessed via panoramic radiography [16] at baseline.

### Sample size

Sample size was calculated using the Epidemiology and Statistics Laboratory (LEE) tool (http://www.lee.dante.br/pesquisa/amostragem/calculo_amostra.html) based on the following parameters: the standard deviation obtained from pain intensity through the visual analogic scale (9.94 mm) [23]; the difference stipulated between groups, which was 5 mm; a significance level of 95%; and a power of 80%. Thus, 17 participants per group were required for the study. For the pilot study, 25% of the sample size was defined, with an additional 10% to account for potential losses, resulting in a requirement of 5 participants per group.

### Statistical methods

Statistical analyses were performed using the statistical package SPSS (Statistical Package for the Social Sciences Inc.) version 26.0. A 95% confidence interval was used, and a 5% level of significance was adopted. The data normal distribution was verified through the Shapiro-Wilk test. The inter- and intra-group comparisons were conducted using the Mann-Whitney and Wilcoxon tests, respectively. For repeated measures, data were subjected to the Friedman test, with Bonferroni post-hoc analysis. Categorical data were evaluated using Fisher’s Exact test.

## Results

**Fig. 1:**
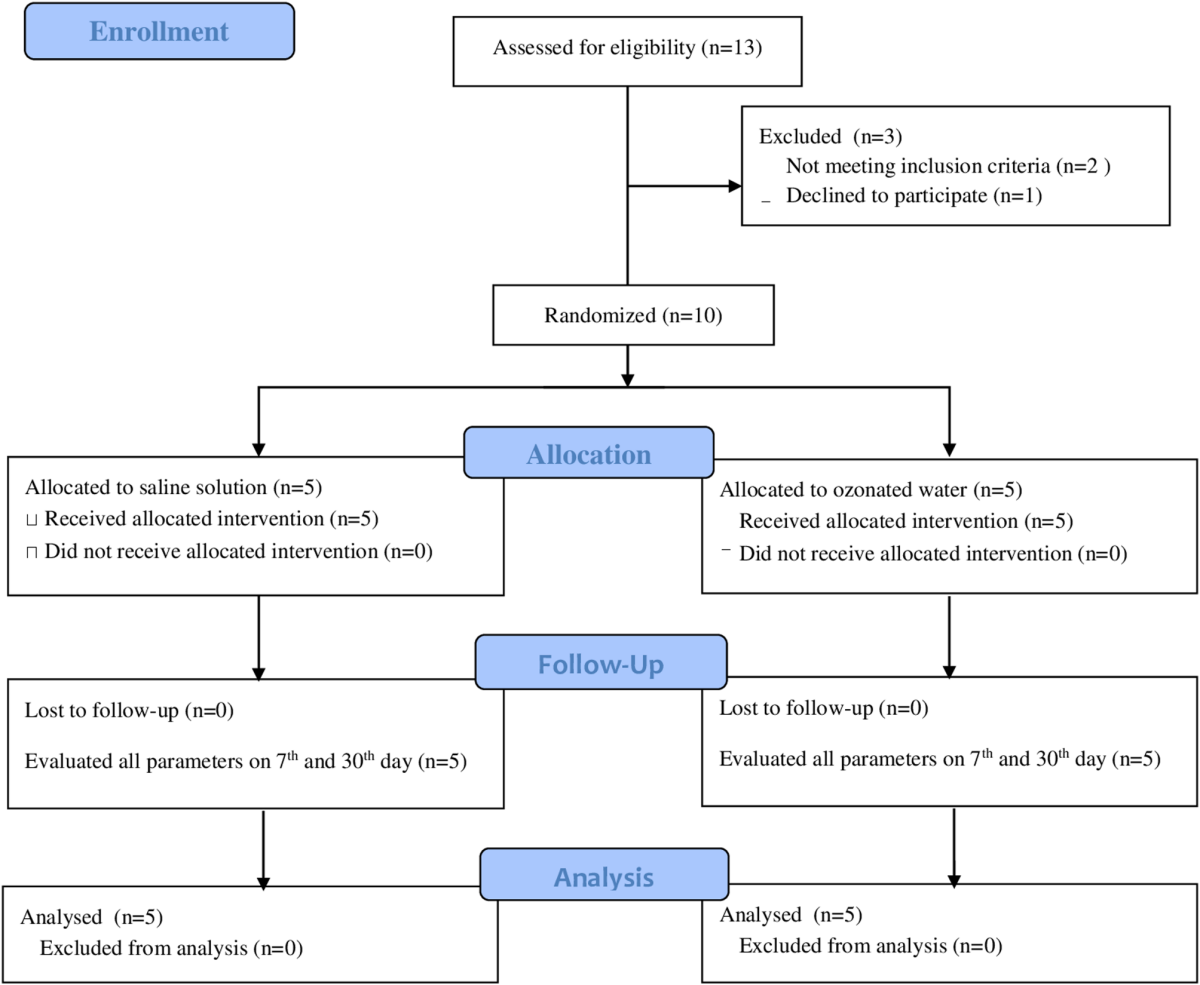
Flow chart depicting the study design

**Fig. 2:**
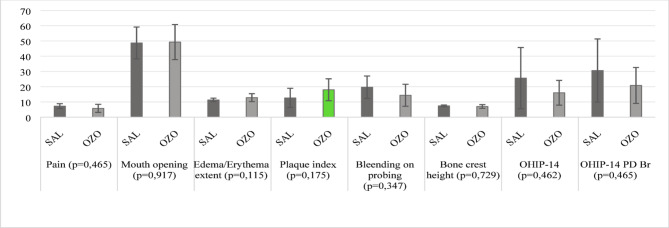
Clinical parameters of groups on *baseline*. The evaluated parameters are presented with the mean, standard deviation, and *p*-value

The number of participants and the reasons for the losses are described in the Flowchart (Fig. 1). Both groups showed similarities regarding sociodemographic data (Table 1) and clinical parameters (Fig. 2). The most frequent position among the teeth included in the study, according to the Pell and Gregory classification, was 2 A (70.0%), followed by 2B (20.0%) and 1 A (10.0%).

**Table 1:** Socio-demographic and outcome characteristics

Variable	*SAL group*		*OZO group*		
	**n**	**%**	**n**	**%**	**p**
Tooth					0,999
38	3	60,0	3	60,0	
48	2	40,0	2	40,0	
Gender					0,490
Female	4	80,0	3	60,0	
Male	1	20,0	2	40,0	
Alcohol					0,500
No	1	20,0	0	0,0	
Yes	4	80,0	5	100,0	
Smoker					0,083
No	5	100,0	2	40,0	
Yes	0	0,0	3	60,0	
Age	25,8 (± 4,02)		25,8 (± 3,06)		0,999
Pell and Gregory Classification					
1 A	1	20,0	0	0,0	0,311
2 A	2	40,0	5	100,0	
2B	2	40,0	0	0,0	

Data (tooth, gender, alcohol, smoker and Pell and Gregory Classification) were shown as absolute (n) and relative (%) frequencies. Age was shown as mean (± standard deviation)

**Fig. 3:**
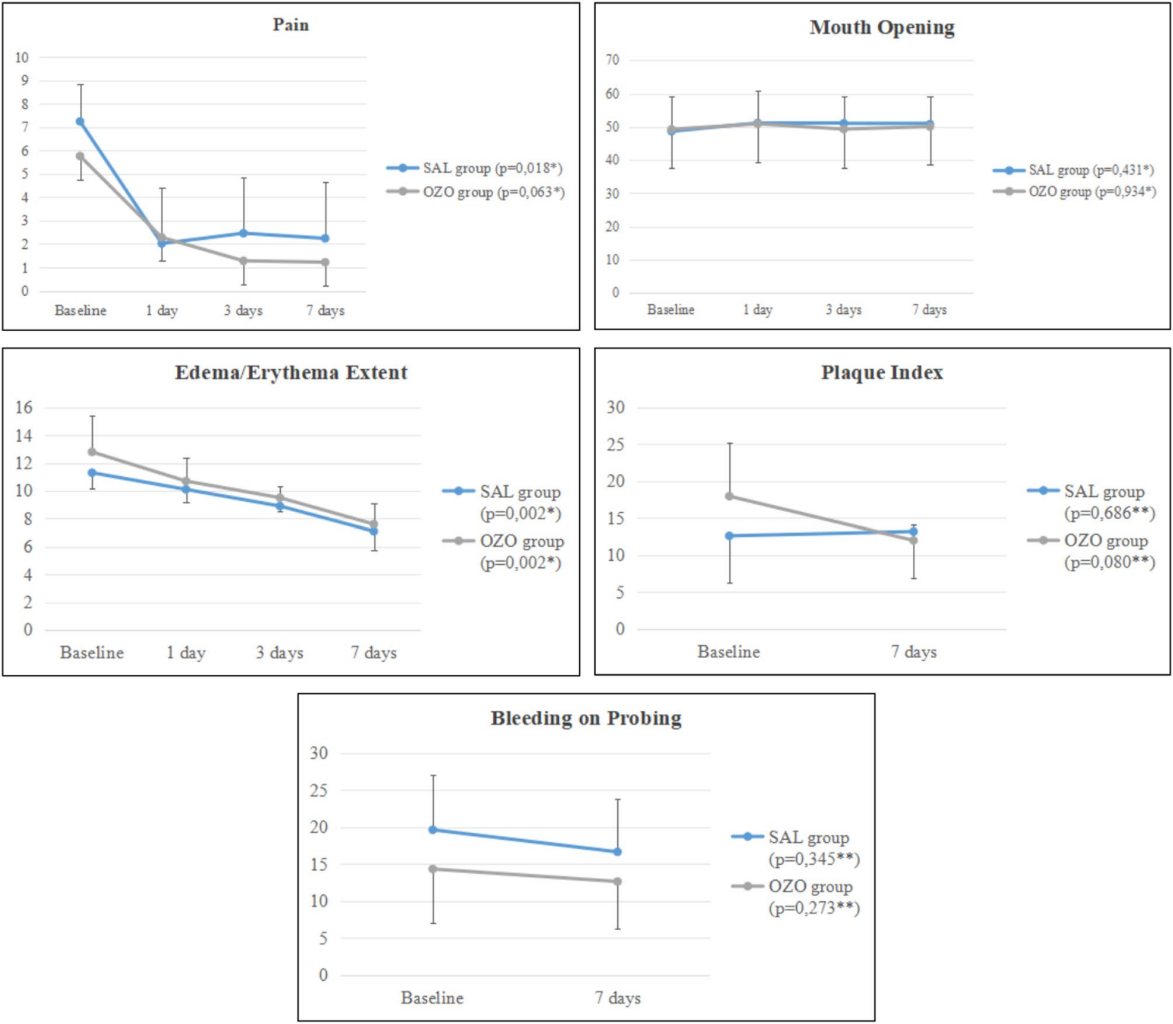
Intragroup analysis of evaluations up to 7 days after treatment. Data (Pain, Mouth Opening, Edema/Erythema Extent, Plaque Index, Bleeding on Probing) were shown as means ± standard deviation, and *p*-value for both groups on baseline, 1, 3, and 7 days post-treatment. ^* Friedman Test, Boferroni post−hoc ** Wilcoxon Test^

There was no statistical difference for the clinical parameters evaluated in the intergroup analysis 7 days after treatment (Table 2). However, in the intragroup analysis, both groups showed improvement in some parameters. The SAL group improved in the parameters of pain and edema/erythema extent (*p* = 0,018; *p* = 0,002; respectively), while the OZO group showed improvement only in the edema/erythema extent (*p* = 0,002), with no improvement in other parameters (Fig. 3).

**Table 2:** Intergroup analysis 7 days after treatment. The evaluated parameters are presented with the mean, standard deviation (SD), and *p*-value for both groups

Variable	*SAL*		*OZO*		
	**Mean**	**SD**	**Mean**	**SD**	**p**
Pain	2,24	2,43	1,22	1,49	0,599
Mouth opening	50,80	8,48	50,08	8,93	0,917
Edema/erythema extent	7,10	1,39	7,60	1,52	0,456
Plaque index	13,20	6,34	12,00	2,10	0,917
Bleending on probing	16,66	7,18	12,65	6,31	0,465

SD: standard deviation

**Fig. 4:**
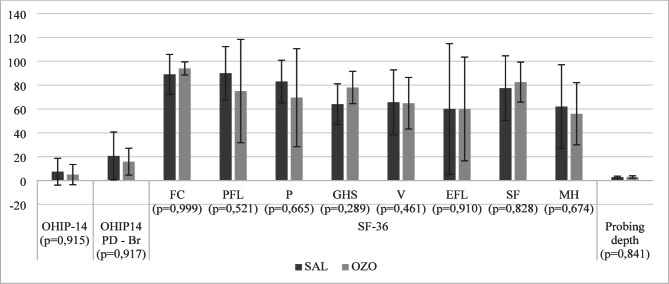
Intergroup analysis of quality of life and probing depth of the 2nd molar, 30 days after treatment

Regarding quality of life and probing depth in the second molar adjacent to the treated tooth in the study, there was no difference in the intergroup comparison (Fig. 4). In the intragroup analysis, there was a difference for the SAL group in OHIP-14 and OHIP-14 PD Br (*p* = 0,043; *p* = 0,042, respectively) and for the OZO group in OHIP-14 and in the GHS domain of the SF-36 (*p* = 0,041; *p* = 0,034; respectively); there was no difference in any of the other SF-36 domains or other evaluated parameters (Table 3).

**Table 3:** Intragroup analysis of alveolar bone crest height, probing depth of the 2nd molar, and quality of life, 30 days after treatment

Variable			*Baseline*		30 days		
			**Mean**	**SD**	**Mean**	**SD**	**p**
SAL group	Bone crest height		7,40	0,55	6,40	0,55	0,059
	Probing depth of the 2nd molar		4,00	0,70	2,80	0,83	0,063
	OHIP14		25,60	20,07	7,40	11,22	**0**,**043**
	OHIP14 PD Br		30,60	20,72	20,60	20,06	**0**,**042**
	SF-36	FC	73,00	27,06	89,00	16,73	0,197
		PFL	55,00	41,08	90,00	22,36	0,066
		P	78,50	22,19	83,00	17,80	0,854
		GHS	61,00	18,17	64,00	17,10	0,257
		RE	61,60	30,80	65,60	27,22	0,102
		EFL	60,00	54,77	60,00	54,77	0,999
		SF	67,50	39,13	77,50	27,10	0,257
		MH	59,00	33,80	62,00	35,11	0,461
OZO group	Bone crest height		7,00	1,22	6,00	0,71	0,102
	Probing depth of the 2nd molar		4,40	0,89	3,00	1,00	0,066
	OHIP14		16,00	8,12	5,00	8,46	**0**,**041**
	OHIP14 PD Br		20,80	11,78	15,80	11,23	0,066
	SF-36	FC	91,00	12,45	94,00	5,48	0,593
		PFL	75,00	43,30	75,00	43,30	0,999
		P	69,00	36,77	69,50	41,09	0,999
		GHS	71,00	15,57	78,00	13,51	**0**,**034**
		RE	56,80	26,89	64,80	21,61	0,104
		EFL	66,66	33,35	60,00	43,47	0,713
		SF	62,50	31,87	82,50	16,77	0,257
		MH	56,00	30,29	56,00	26,08	0,999

SD: standard deviation. The domains assessed by the SF-36 are FC = Functional capacity; PF = Physical functioning limitations; P = Pain; GHS = General health status; RE = Vitality; EF = Emotional functioning limitations; SF = Social functioning; MH = Mental health

## Discussion

This study is the first to provide experimental evidence of the effects of ozonated water on clinical parameters and quality of life in patients with lower third-molar pericoronitis. Results suggest that ozonated water improves certain clinical parameters, such as pain and edema/erythema extent, as well as patient quality of life. The study aimed to investigate whether ozonated water treatment as an adjunct improves clinical outcomes and quality of life compared to saline solution treatment, hypothesizing potential patient improvement.

The study hypothesis was partially accepted, as the use of ozonated water as an adjunct to treatment improved pain, edema/erythema extent, and quality of life for patients; however, there was no influence on improving other clinical parameters. This may be related to the sample size, as being a pilot study, it was not of significant size to statistically significant differences between parameters [24, 25].

Previous studies have explored new alternatives for pericoronitis treatment, ranging from novel therapies to comparisons between different drugs. However, when assessing patient pain, none of them showed significant differences compared to the control group [23, 26–28]. Mouth opening, also assessed in some studies as another manifestation related to pericoronitis, showed no significant difference between the study groups [23, 27, 28].

The improvement in pain and edema/erythema extent, evaluated 7 days after treatment, may be related to the early stage of inflammation in the study patients, where symptoms such as pain and local swelling are more noticeable. Additionally, the possibility of an early stage of inflammation may also explain the lack of perceived influence on mouth opening, plaque index, and bleeding on probing, as the pre-treatment time was not sufficient for inflammation to spread to surrounding regions, with no systemic changes observed in the patients [29]. These factors may be related to the quality of patient’s oral hygiene, as cases of less acute pericoronitis are more common in patients with good or moderate oral hygiene [30], as well as related to the “Hawthorne Effect,” where patients behave differently due to being evaluated. The improvement in the edema/erythema extent for clinical use represents an enhancement in patient comfort and a possible reduction in inflammation.

It is known that mechanical debridement contributes to reducing plaque index and bleeding on probing, as evidenced by studies [31, 32], thereby improving patients’ health status and, consequently, their quality of life. Additionally, the improvement in quality of life observed in both groups through the OHIP-14 questionnaire assessment may be related to the reduction in clinical parameters and, consequently, the improvement in patients’ inflammatory conditions.

Previous studies have shown that pericoronitis treatment, either through periodontal care or third molar extraction, improves patients’ quality of life, using the same quality of life assessment questionnaire as this study [6, 32–35]. There was no significant difference in intergroup evaluation, suggesting that the results of ozonated water are similar when used as an adjunct in pericoronitis treatment. An important finding of the study is the improvement in the quality of life of control patients when using a specific periodontal disease questionnaire [18], possibly related to the improvement in their clinical parameters.

Despite the importance of pilot studies in assessing the feasibility of methodologies for main studies, they cannot be relied upon solely to determine the significance of findings in clinical studies [36]. However, they serve as models for methodological improvements in future studies and sample size calculation [24, 37]. Therefore, studies with larger samples should be conducted to seek clarification for these inquiries.

Methodologically, ensuring equality in baseline characteristics between study groups is crucial to avoid initial differences that could confound comparisons throughout the research. Moreover, it becomes a critical factor in ensuring that results can be attributed to the treatment or intervention under study rather than initial group differences [38].

### Limitations

It’s challenging to assess various clinical parameters and achieve significant differences using a pilot study methodology, even if it’s triple-blinded and with low chances of biases. Furthermore, the contact time of ozonated water with pericoronitis was too short, considering it only lasted during irrigation treatment. Therefore, studies with larger sample sizes and good methodological quality should be conducted.

Additionally, different groups should be added to the methodology, some with alternatives (such as rinsing with ozonated water after a certain treatment period or using ozonated oil) that allow the affected area to have more contact time with the ozonated substance. Also, challenging patients should be included, such as smokers and diabetics, in whom ozonated water may provide additional benefits.

### Clinical implication

In summary, this study proposed a new adjunctive treatment approach for pericoronitis, aiming to compare it with standard treatment in improving clinical parameters and quality of life. The results demonstrated that ozonated water showed similar effects to saline solution, with both also improving patient quality of life. Translating these findings to routine clinical practice, it can be speculated that ozonated water is an alternative irrigant for debridement treatments of pericoronitis cases.

## Conclusion

Adjunct treatment with ozonated water or saline solution led to clinical improvement and enhanced quality of life in pericoronitis patients, with no difference between the groups. Therefore, the use of ozonated water can be considered an effective alternative in pericoronitis treatment, although further research is needed to gain a comprehensive understanding of its benefits and potential applications in dentistry.

## Electronic supplementary material

Below is the link to the electronic supplementary material.

Supplementary Material 1

## Data Availability

The datasets used and/or analysed during the current study are available from the corresponding author on reasonable request.
