# Supplementary material for: Ozonated water to treat pericoronitis - insights from a randomized triple-blind pilot trial
Source: BMC Oral Health. 2025 Jul 28;25:1271. doi: 10.1186/s12903-025-06652-5 (PMC12302791; doi:10.1186/s12903-025-06652-5)
Supplement: Supplementary file 1 — Supplementary Material 1 [file 12903_2025_6652_MOESM1_ESM.docx]

**MATERIALS AND METHODS**

**Study Design and Ethical Principles**

This study was designed as a randomized, controlled, triple-blind clinical trial with a follow-up period of up to thirty (30) days involving patients diagnosed with pericoronitis in the lower third molar. The sample consisted of participants diagnosed with symptomatic pericoronitis at the Periodontology and Surgery Clinic of the Federal University of the Jequitinhonha and Mucuri Valleys (UFVJM).

Participants who accepted the invitation to take part in the clinical trial were randomly assigned to two groups and received the proposed emergency treatment. Prior authorization was obtained from the Department of Dentistry to access and conduct the study within the clinic. The study’s experimental design followed the CONSORT (Consolidated Standards of Reporting Trials) checklist (Schulz et al., 2010).

The research project was submitted to the UFVJM Research Ethics Committee (CEP) and approved under opinion number 5.922.185. In line with the ethical principles established in this resolution, all participants received a presentation letter explaining the main aspects of the research. Only those who agreed to participate and signed the Informed Consent Form were included in the study. This clinical trial was registered at the Brazilian Clinical Trials Registry (http://www.ensaiosclinicos.gov.br/rg/RBR-79pss6w/) on 22/11/2024.

**Training and Calibration**

Training and clinical calibration were conducted to measure clinical parameters, analyze radiographs, and classify the positions of third molars. A single researcher (CLFS) was calibrated at the UFVJM Periodontology and Surgery Clinic using 10 panoramic radiographs (from patients not involved in the study) via a test-retest protocol with a 15-day interval. The intraclass correlation coefficient (kappa) was 0.9. The same researcher was also calibrated on the correct technique for measuring all clinical parameters.

**Sample Size Calculation**

The sample size was calculated using the Epidemiology and Statistics Laboratory (LEE) tool (<http://www.lee.dante.br/pesquisa/amostragem/calculo_amostra.html>), considering a pain score standard deviation of 9.94 mm (based on visual analog scale data) and a between-group difference of 5 mm. A significance level of 5% and test power of 80% were set. Based on this, 15 participants per group were required, with an additional 10% to account for dropouts, totaling 17 participants per group (Shahakbari et al., 2014), or 34 participants overall. As a pilot study, 25% of the total sample was designated for preliminary testing, plus 10% for losses, resulting in 10 participants (5 per group).

**Recruitment**

Participants were recruited among individuals who spontaneously sought treatment at the UFVJM dental school clinics during routine care and who voluntarily agreed to participate after being diagnosed with symptomatic pericoronitis. Additionally, the study was advertised via social media (Instagram, WhatsApp, Facebook), providing immediate treatment and the researcher's contact information.

**Inclusion Criteria**

Participants were eligible if they met the following conditions: (1) signs/symptoms of pericoronitis such as spontaneous pain, erythema, or purulent drainage affecting the gingiva over the lower third molar; (2) age between 18 and 35 years; (3) ASA I or ASA II surgical risk classification; (4) periodontal health or levels I and II periodontal status, according to the American Academy of Periodontology; and (5) signed informed consent (ICF).

**Exclusion Criteria**

Exclusion criteria included: (1) antibiotic use within the previous two months; (2) any medical contraindication for periodontal probing; (3) contraindication to analgesics; (4) contraindication to ozone therapy.

**Randomization, Blinding, and Allocation Concealment**

As a triple-blind clinical trial, the following procedures ensured participant, operator, and evaluator blinding: (1) both treatment liquids were packaged identically in terms of size, shape, and volume, labeled with codes revealed only after data analysis; (2) the liquids were both clear, odorless, and tasteless, preventing visual or sensory identification by researchers or patients; (3) data collection sheets were standardized and coded; (4) treatment assignment was randomized via opaque envelopes, each containing five slips labeled "A" or "B," drawn during the treatment session by someone unaffiliated with the study; (5) researchers had distinct roles: one administered clinical assessments, one prepared and packaged the treatment solutions, and another performed the treatments.

To verify the blinding, all participants were asked post-treatment—by an independent individual—whether they could identify the product used. None of the participants were able to identify their treatment.

**Procedures**

**Clinical Procedures**

For patients with systemic symptoms (e.g., fever, malaise, lymphadenopathy), antibiotics were prescribed one hour before treatment for a 7-day course. Amoxicillin 500 mg every 8 hours was prescribed; clindamycin 300 mg every 8 hours was used for allergic individuals.

Local anesthesia was administered using 2% lidocaine with epinephrine 1:100,000 (Lidocaine 2% DFL®). Treatment involved debridement under the pericoronal flap using gauze and curettes, followed by irrigation with the randomly assigned solution. Irrigation was performed using a standardized 20 mL volume via a syringe: saline solution (GROUP A) or ozonated water (GROUP B).

Participants received oral hygiene instructions and a standardized hygiene kit. Follow-ups occurred at 1, 3, 7, 15, and 30 days post-treatment. Afterward, patients were referred for (1) extraction if there was insufficient space for eruption, (2) distal wedge surgery if excess distal gingiva was present despite available space, or (3) monitoring if eruption occurred without excessive gingiva.

**Ozonated Water Production**

Ozonated water was prepared 5 minutes before use by an external researcher (Ivanete Docarmo Ribeiro Moreira) using MilliQ water (provided by CIPq Lab - UFVJM). A MedPlus V ozone generator (Philozon®, Santa Catarina, Brazil), fitted with a glass column and stainless steel microbubble diffuser, was used.

The generator was set to 60 μg/mL and operated for five continuous minutes to ozonate 250 mL of MilliQ water.

**Outcomes, Timepoints, and Evaluation Instruments**

Primary outcomes included pain and quality of life. Secondary outcomes were probing depth and alveolar crest height distal to the second molar, mouth opening, and edema/erythema extension measured by lesion diameter. Tables 1 and 2 list the evaluation instruments and time points for each outcome.

**Primary Outcomes**

| **Table 1.** Parameters, assessment tools, and evaluation times for primary outcomes. | | |
| --- | --- | --- |
| **Parameter** | **Instrumento de avaliação/referência** | **Tempos de avaliação** |
| Pain | Escala visual analógica (EVA) | Baseline, 1, 3, 7, 15 and 30 days post-treatment |
| Quality of Life | Oral Health Impact Profile (OHIP-14)  Short Form 36 (SF-36)  OHIP 14 PD-Br | Baseline and 30 days post-treatment |

**Secondary Outcomes**

| **Table 2.** Parameters, assessment tools, and evaluation times for secondary outcomes. | | |
| --- | --- | --- |
| **Parameter** | **Assessment Tool** | **Evaluation Times** |
| Mouth Opening | Millimeter ruler | Baseline, 1, 3, 7, 15 and 30 days post-treatment |
| Edema/Erythema Extension | Dental floss and transfer to a millimeter ruler | Baseline, 1, 3, 7, 15 and 30 days post-treatment |
| Visible Plaque Index | WHO Probe | Baseline, 7, 15, and 30 days post-treatment |
| Bleeding on Probing Index | WHO Probe | Baseline, 7, 15, and 30 days post-treatment |
| Probing Depth | Computerized pressure-controlled probe (Florida Probe Corporation, Gainesville, FL, USA) | Baseline, 7, 15, and 30 days post-treatment |
| Alveolar Crest Height | Williams probe and measurement using a digital caliper in millimeters | Baseline and 30 days post-treatment |
| 3^rd^ Molar Position | Panoramic radiograph | Baseline |

**Evaluated Parameters**

**Pain**

Pain was measured at baseline and at 1, 3, 7, 15, and 30 days after immediate treatment using the Visual Analog Scale (VAS) for pain. This is a unidimensional instrument for assessing pain intensity, validated and widely used among dental professionals and researchers (Wilson et al., 1994; Magnusson et al., 1995).

The VAS consists of a 10-centimeter (cm) line with endpoints marked by vertical bars: one end labeled "no pain" and the other "worst pain imaginable." Participants were asked to evaluate and mark their current pain level on the line. The distance from the “no pain” endpoint to the marked point was measured using a millimeter ruler, and the value was recorded.

In addition, the number of analgesic tablets taken was recorded by the patient up to the seventh postoperative day. All participants received a prescription for 500 mg sodium dipyrone every 6 hours, to be taken in case of pain. No participant reported an allergy to the prescribed medication, and therefore, no alternative prescription was needed.

**Quality of Life (QoL)**

Quality of life was assessed using three questionnaires: the Oral Health Impact Profile (OHIP-14), the Short Form-36 (SF-36), and the Oral Health Impact Profile for Periodontal Disease (OHIP-14 DP-Br), applied at baseline and after 30 days (Appendices IV, V, and VI, respectively).

The OHIP-14 is a simplified version of the original 49-item OHIP-49 questionnaire. It was translated and validated for use in Brazil (Oliveira et al., 2005) and includes 14 questions across seven domains: functional limitation, physical pain, psychological discomfort, physical disability, psychological disability, social disability, and social disadvantage. Responses are scored from 0 to 4, yielding a maximum total score of 56.

The SF-36 is a general health-related quality-of-life questionnaire developed in the United States (Ware et al., 2003). It has demonstrated good sensitivity in various settings and addresses limitations of skewed response distributions (e.g., too many "excellent" or "very poor" ratings). It has been translated and validated for the Brazilian population, proving suitable for local socioeconomic and cultural conditions (Ciconelli et al., 1999), and was therefore selected to assess general health-related QoL in this study.

The OHIP-14 DP-Br, developed in Mexico and culturally adapted and validated for Brazilian Portuguese (Cruz, 2023), is a version of the OHIP specifically targeting periodontal disease. It includes 14 questions covering seven domains, scored on a 5-point scale (0–4), with a maximum score of 56.

**Mouth Opening (MO)**

Maximum mouth opening was measured using a millimeter ruler, following the methodology described by Üstün et al. (2003), at baseline and at 1, 3, 7, 15, and 30 days post-treatment.

Participants were seated with feet flat on the floor, spine erect, and head positioned according to the Frankfurt horizontal plane and the median sagittal plane perpendicular to the horizontal. They were instructed to open their mouths as wide as possible without assistance. The distance between the incisal edges of the upper and lower right central incisors was measured three times using a millimeter ruler, and the average value was recorded in millimeters.

**Edema/Erythema Extension (EEE)**

The extent of edema/erythema was assessed by measuring the maximum vestibulo-lingual and mesio-distal dimensions of the lesion using dental floss. These measurements were then transferred to a millimeter ruler, and the average of the two distances was calculated to determine the mean diameter of the local edema/erythema. Evaluations were performed at baseline and at 1, 3, 7, 15, and 30 days post-treatment (Alcantara et al., 2014).

**Visible Plaque Index (VPI)**

The dichotomous Visible Plaque Index (O’Leary et al., 1972) was used to assess the presence or absence of dental biofilm on four surfaces of each tooth. The result was expressed as the percentage of surfaces with plaque and was evaluated at baseline and at 1, 3, 7, 15, and 30 days post-treatment.

**Bleeding on Probing Index (BPI)**

The dichotomous Bleeding on the Probing Index (Ainamo & Bay, 1975) was determined by the presence or absence of bleeding within 15 seconds of probing on four surfaces of each tooth using a WHO periodontal probe. Assessments were conducted at baseline and at 1, 3, 7, 15, and 30 days post-treatment.

**Probing Depth (PD)**

Probing depth was measured from the gingival margin to the base of the sulcus or periodontal pocket adjacent to the second molar anterior to the treated third molar. Six sites per tooth were examined—three on the buccal and three on the lingual surface—using a computer-assisted, controlled-force probe (Florida Probe Corporation, Gainesville, FL, USA) (Gibbs et al., 1988). Measurements were taken at baseline and at 7, 15, and 30 days post-treatment.

**Alveolar Crest Height (ACH)**

Alveolar crest height was measured from the marginal crest to the alveolar bone crest under local infiltrative anesthesia. A Williams periodontal probe, adapted with a rubber marker, was used, and measurements were taken in millimeters using a digital caliper. This parameter was assessed at baseline and 30 days after treatment.

**Third Molar Position**

The position of the lower third molar was classified according to the Pell & Gregory classification (1933). For this, panoramic radiographs were taken at baseline only.

**Data Collection and Recording**

Data were collected using questionnaires administered prior to dental care, including a clinical-epidemiological form designed to capture variables such as place of origin, sex, race, age, marital status, occupation, and habits, among others. This form was adapted from those used in UFVJM’s dental school clinics (Appendix VII). Socioeconomic and educational information was obtained from an adapted version of the SBBrasil 2010 survey (Appendix VIII).

Pain scores, the number of analgesics used, and clinical parameters were also recorded using specific forms (Appendix I).

**Statistical Analysis**

Statistical analyses were performed using SPSS® software (Statistical Package for the Social Sciences Inc., Version 26.0, New York, USA). Descriptive statistics included frequency, mean, and standard deviation. The Shapiro-Wilk test was used to assess normality.

Between-group and within-group comparisons were made using the Mann-Whitney and Wilcoxon tests, respectively. For repeated measures, the Friedman test was applied, followed by the Bonferroni post-hoc test. Categorical data were analyzed using Fisher’s exact test. A significance level of 5% (p < 0.05) was adopted.
